# Renal Perfusion and Function during Pneumoperitoneum: A Systematic Review and Meta-Analysis of Animal Studies

**DOI:** 10.1371/journal.pone.0163419

**Published:** 2016-09-22

**Authors:** Kimberley E. Wever, Moira H. D. Bruintjes, Michiel C. Warlé, Carlijn R. Hooijmans

**Affiliations:** 1 Systematic Review Centre for Laboratory animal Experimentation (SYRCLE), Radboud university medical center, Nijmegen, The Netherlands; 2 Department of Surgery, Radboud university medical center, Nijmegen, The Netherlands; University Hospital Oldenburg, GERMANY

## Abstract

Both preclinical and clinical studies indicate that raised intra-abdominal pressure (IAP) associated with pneumoperitoneum during laparoscopic surgical procedures can cause renal damage, the severity of which may be influenced by variables such as pressure level and duration. Several of these variables have been investigated in animal studies, but synthesis of all preclinical data has not been performed. This systematic review summarizes all available pre-clinical evidence on this topic, including an assessment of its quality and risk of bias. We performed meta-analysis to assess which aspects of the pneumoperitoneum determine the severity of its adverse effects. A systematic search in two databases identified 55 studies on the effect of pneumoperitoneum on renal function which met our inclusion criteria. There was high heterogeneity between the studies regarding study design, species, sex, pressure and duration of pneumoperitoneum, and type of gas used. Measures to reduce bias were poorly reported, leading to an unclear risk of bias in the majority of studies. Details on randomisation, blinding and a sample size calculation were not reported in ≥80% of the studies. Meta-analysis showed an overall increase in serum creatinine during pneumoperitoneum, and a decrease in urine output and renal blood flow. Subgroup analysis indicated that for serum creatinine, this effect differed between species. Subgroup analysis of pressure level indicated that urine output decreased as IAP level increased. No differences between types of gas were observed. Data were insufficient to reliably assess whether sex or IAP duration modulate the effect of pneumoperitoneum. Four studies assessing long-term effects indicated that serum creatinine normalized ≥24 hours after desufflation of pneumoperitoneum at 15mmHg. We conclude that harmful effects on renal function and perfusion during pneumoperitoneum appear to be robust, but evidence on long-term effects is very limited. The reliability and clinical relevance of these findings for healthy patients and patients at high risk of renal impairment remain uncertain. We emphasize the need for rigorous reporting of preclinical research methodology, which is of vital importance for clinical translation of preclinical data.

## Introduction

Laparoscopic surgery is rapidly replacing the open approach in many fields of surgery. Although laparoscopic procedures have many advantages (*e*.*g*. improved early postoperative recovery and cosmetic benefit), a number of studies have reported adverse effects of the required pneumoperitoneum, *i*.*e*. the insufflation of gas or air into the abdominal cavity, in order to create working space between the abdominal wall and the intra-abdominal organs. Both preclinical and clinical studies indicate that raised intra-abdominal pressure (IAP) during laparoscopic procedures can affect several homeostatic systems, causing alterations in cardiovascular, pulmonary and renal physiology [1]. The renal effects have been attributed to a number of factors, including compression of the renal vein and parenchyma, increased total renal vascular resistance, increased antidiuretic hormone production, and secondary effects of decreased cardiac output [1]. In patients with no or limited co-morbidity, disturbances in renal function due to pneumoperitoneum are often transient and clinically of little importance. However, in patients with pre-existing renal dysfunction, pneumoperitoneum may cause further deterioration. In addition, in procedures such as laparoscopic live donor nephrectomy and (partial) tumour nephrectomy, it is of great importance to preserve function of the remaining kidney. It is therefore important to identify determinants of the harmful effects of pneumoperitoneum, in order to minimize damage whenever possible.

One important variable which influences the severity of pneumoperitoneum-induced adverse effects is the intra-abdominal pressure (IAP) level. A recent review of the clinical evidence for the use of high *versus* low pressure pneumoperitoneum concluded that the available evidence on the effects on renal function was very limited and of moderate quality [2]. Other possible determinants, such as pneumoperitoneum duration, the type of gas used and patient-related characteristic such as sex have not been (extensively) studied in patients yet. Several of these variables have been investigated in preclinical animal studies (e.g. [3–5]), but a systematic review including synthesis of all preclinical data has not been performed. This systematic review provides insight in all available pre-clinical evidence on the effects of pneumoperitoneum on renal function, including an assessment of its quality and risk of bias. We performed meta-analysis to investigate which aspects of the pneumoperitoneum modulate these effects, in order to provide guidance for the optimization of the use of pneumoperitoneum during laparoscopic procedures with regard to *e*.*g*. the IAP level, IAP duration, and the type of gas used.

## Materials and Methods

The review methodology was specified in advance and documented using SYRCLE’s systematic review protocol for animal intervention studies (see S1 File and [6]).

### Amendments to the review protocol

In order to further optimize the quality assessment, we assessed three additional study quality indicators not prespecified in the protocol: reporting any blinding, regulation of body temperature within 3°C variation and reporting of a sample size calculation.

We aimed to perform sensitivity analysis to assess the effect of studies with low methodological quality on the meta-analysis results by excluding these studies and repeating the analysis. However, this analysis was omitted since the quality of the studies was too low to sensibly stratify studies by quality.

Schaeffer 2012 was the only study measuring baseline data during laparotomy. These data were pooled in the overall analysis, but we performed sensitivity analysis by excluding these data to check their effect on analysis outcome.

### Literature search strategy

We performed a systematic, computerized search in PubMed and EMBASE to identify all animal studies comparing renal function with and without elevated IAP induced by pneumoperitoneum. The full search strategy (see S2 File) was based on the search components “animal”[7, 8], “laparoscopic surgery” or “abdominal pressure”, and “renal function”. Search results from each database were combined and duplicates removed. In addition, we checked the reference lists of all included studies and relevant reviews identified by our search for additional eligible references. The search was performed on August 22^nd^ 2013 and updated on February 24^th^ 2015 (the search update yielded 2 additional included references).

### Study selection

Study selection was performed by two independent reviewers (MB and KW or CH) in two phases: 1) screening for eligibility based on title and abstract and 2) final inclusion based on full-text assessment. Disagreements were solved by discussion with a fourth investigator (MW). Studies were included if they met all of the following criteria: 1) the study was an original full paper which presented unique data; 2) the study was performed in animals *in vivo;* 3) the study compared the effect of increased IAP due to pneumoperitoneum *versus* no IAP; and 4) the study reported on the outcome measures serum creatinine, renal blood flow, urine output and/or renal histology. To avoid heterogeneity between renal histology scores[9], studies reporting this outcome were included only if Jablonski’s renal damage score was used [10]. No language or publication date restrictions were applied. If necessary, publications in languages other than English were translated by a native speaker for that particular language.

### Study characteristics and data-extraction

We extracted bibliographic details such as author, journal and year of publication, as well as data on the following study characteristics: animal species, strain, sex, age, weight, level of IAP in experimental and control groups, type of gas used for insufflation, duration of IAP, timing of the outcome measurement and the type of control data. Regarding the latter, we included studies comparing data from animals undergoing pneumoperitoneum to a separate control group without pneumoperitoneum, as well as studies which compared data during or after pneumoperitoneum to baseline measurements performed beforehand in the same animals (referred to as Δ baseline studies in this paper).

We aimed to extract outcome data for serum creatinine, renal blood flow, urine output and renal histology assessed by Jablonski scale. For renal blood flow, we included comparable outcomes such as renal artery flow, renal vein flow, renal cortical perfusion and renal medullary perfusion. Data were extracted if the mean, standard deviation (SD) and number of animals (n) were reported, or could be calculated, for the control and experimental group(s). If the standard error of the mean (SEM) was reported, it was converted to SD for meta-analysis. For twenty articles, not all relevant outcome measures or study details could be extracted. We therefore contacted these authors via e-mail and received response from fifteen authors, of which fourteen provided additional data.

If a study applied a range of increasing IAP levels in the same animal, we extracted the data obtained at baseline and at the first IAP level only, since the data obtained at subsequent IAP levels may be affected by previous levels.

### Study quality and risk of bias assessment

Two reviewers (MB, KW or CH) independently assessed the risk of bias and study quality of each included study. In case of discrepancies, consensus was reached through discussion. Risk of bias was assessed using SYRCLE’s Risk of Bias tool[11]. When assessing selection bias, groups within a study were considered similar at baseline if strain, sex and body weight or age did not differ significantly between groups. To assess whether studies were free of other risks of bias, we took into account the application of a Veress needle in both the control and experimental groups and possible conflicts of interest. In addition to the risk of bias assessment, we also assessed reporting of the following study quality indicators: any randomization, any blinding, regulation of body temperature within 3°C variation and sample size calculation. For Δ baseline studies, criteria concerning randomization were considered not applicable to the study design.

### Data synthesis and meta-analysis

A minimum of 5 studies reporting an outcome measure was required to perform meta-analysis. We aimed to perform separate meta-analyses for data obtained during pneumoperitoneum, and long-term outcome data (obtained ≥24 hours after desufflation of the pneumoperitoneum). The number of studies reporting data obtained at long-term time-points was too limited to perform reliable meta-analysis. Their results are therefore reported in a descriptive summary.

Meta-analysis for data obtained during pneumoperitoneum was performed using Comprehensive Meta-Analysis (version 2.2.064, Biostat Inc., Englewood, USA) and STATA (version 11.2, StataCorp, Texas, USA). For the outcome measure serum creatinine this was done by computing the raw difference in means (MD; experimental group mean minus control group mean) in mg/dl for all studies. Meta-analysis of the outcome measures urine output and renal blood flow was performed by computing the standardized difference in means (SMD; experimental group mean minus control group mean, divided by the SD), to account for differences in the units of measurement.

Data were pooled using a random effects model in all analyses to account for anticipated heterogeneity. Effect estimates are reported as MD or SMD with the corresponding 95% confidence intervals. Heterogeneity was assessed and reported as the I^2^ statistic. We used meta-regression (function metareg in STATA) to assess the proportion of variance in the outcome measure explained the subgroup variables.

For serum creatinine we aimed to assess publication bias by examining funnel plot asymmetry if the analysis contained at least 20 comparisons. We did not assess publication bias for outcome measures analyzed as SMD, since funnel plots may become skewed when using this effect measure, leading to unreliable results.

We expected a fundamental difference between control measurements obtained at baseline *versus* measurements obtained from a separate control group, because the duration of anaesthesia at the time of measurement may differ severely. We therefore performed separate meta-analyses for these two types of study design.

We corrected for repeated use of the same control group by dividing the number of animals in the control group by the number of comparisons with this group. If a study reported data for more than one time point, we pooled data of different time points in the overall analyses to correct for repeated measurements of dependent variables.

### Subgroup and sensitivity analyses

Subgroup analyses were predefined and performed to explore possible causes for heterogeneity and to assess which variables influence the effects of pneumoperitoneum on renal function. A minimum of three comparisons per subgroup was required for analysis. The five subgroup variables were: animal species, gender, level of IAP during pneumoperitoneum, type of gas used for inflation, and duration IAP. For the IAP level, we divided studies into three subgroups: low pressure (1–9 mmHg), medium pressure (10–15 mmHg) and high pressure (16–30 mmHg). The medium pressure range was based on the level of pressure used in clinical practice, while the other groups will assess the effect of lowering or raising this pressure. For the IAP duration, we divided the studies into five subgroups, with the duration doubling in each category: 1–30 minutes, 31–60 minutes, 61–120 minutes, 121–240 minutes. Durations >240 minutes were binned in a fifth category. For each outcome measure, the significance level for subgroup analyses was adjusted for the number of analyses using the Bonferroni-Holm method.

We performed sensitivity analyses to investigate whether our study methodology influenced the results of our meta-analysis. As pre-specified, we tested the robustness of our findings for the subgroup variable IAP level by altering the chosen cut-off points for the three categories: for low pressure, 1–9 mmHg was adjusted to 1–10 mmHg; for medium pressure 10–15 mmHg was adjusted to 11–14 or 11–15 mmHg; for high pressure, 16–30 mmHg was adjusted to 15–30 mmHg. For the subgroup variable pneumoperitoneum duration, we altered the chosen five categories to three categories based on equal durations: 1–90 minutes, 91–180 minutes and >180 minutes.

## Results

### Study selection and characteristics

**Fig 1:**
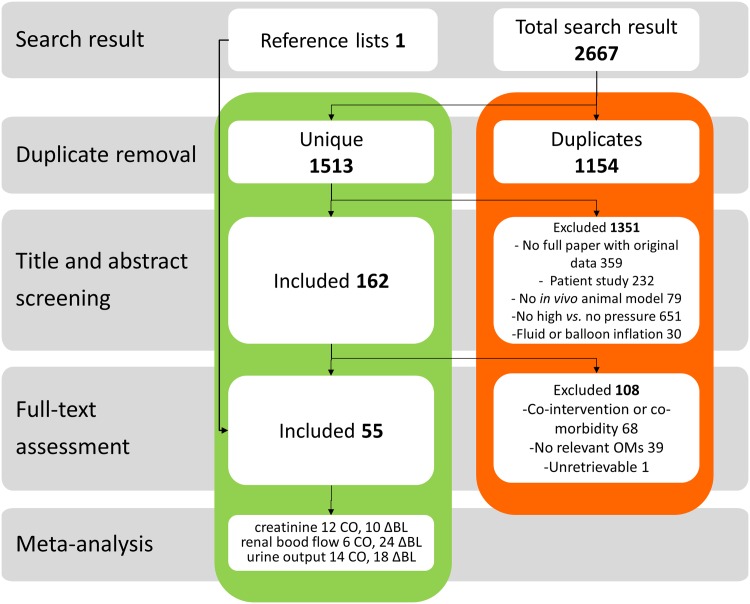
Flow chart of study selection process. The number of studies in each phase is indicated in bold font. OMs = outcome measures; CO = separate control group; ΔBL = repeated measures design comparing data during pneumoperitoneum with control measurements taken at baseline in the same animals.

A flow chart of the study selection process is depicted in Fig 1. The electronic search strategy retrieved 2267 records in total, 1513 of which were unique. After screening on title and abstract, 162 publications met our selection criteria. One additional study was selected for full-text assessment after hand-searching the reference lists of relevant reviews. After full-text assessment, 55 publications were included, two of which were published in Chinese [12, 13].

The characteristics of the included studies are presented in Table 1. As expected, the type of control measurements used differed between studies: twelve studies used a separate control group without pneumoperitoneum, and twenty-four Δ baseline studies were identified. The remaining nineteen studies obtained control measurements both at baseline, as well as from a separate control group. There was a large variation in population and intervention characteristics. Twenty-five studies (45%) were performed in rats, twenty-three (42%) in pigs and the remaining studies used dogs, rabbits or mice. Twenty-three studies (42%) used only male animals, twelve studies (22%) used only females, eight studies (15%) used animals of both sexes and the remaining twelve studies (22%) did not report the sex of the animals. A large variety of intra-abdominal pressures was used in the experimental groups, ranging from 4 to 30 mmHg. In 46 studies (84%) carbon dioxide (CO_2_) was used to establish the pneumoperitoneum. In the remaining experiments, helium, nitrogen, argon, oxygen or (medical) air was used.

**Table 1:** Primary study characteristics.

study ID	type of control data	species	sex	IAP in experimental group(s) (mmHg)	type of gas	outcome measures
Abassi2008^#^ [14]	BL	rat	m	14	CO_2_	uo, rbf
Ali2005 [15]	BL	pig	NR	15	CO_2_	rbf
Almeida2004^#^ [16]	BL	dog	m/f	10	CO_2_	rbf
Avraamidou2012^#^^§^ [17]	BL+CO	pig	f	30	He	cr
Bayar2006^†^ [18]	BL+CO	rabbit	NR	12	CO_2_	cr
Beduschi1999^#^^†^ [19]	BL+CO	rat	NR	15	CO_2_	cr
Benninger2012 [20]	BL+CO	pig	m	20/30	CO_2_	uo
Bergman2006^#^ [21]	BL	pig	f	12	CO_2_	rbf
Bishara2009 [22]	BL	rat	m	7	CO_2_	uo, rbf
Bishara2010^#^ [23]	BL	rat	m	7/10/14	CO_2_	uo, rbf
Bishara2011 [24]	BL	rat	m	7	CO_2_	uo, rbf
Bishara2012 [25]	BL	rat	m	7	CO_2_	uo, rbf
Borba2005^§^ [26]	BL+CO	dog	NR	15	CO_2_	uo
Brundell2002 [3]	BL	pig	f	4/8/12	CO_2_/He	rbf
Carmona2008 [4]	BL+CO	dog	NR	15	CO_2_/He	cr, uo
Chiu1994 [27]	BL	pig	NR	5	NR	rbf
Chiu1995 [28]	BL	pig	NR	15	CO_2_	uo,rbf
Chiu1996^#^ [29]	BL	pig	f	15	CO_2_	cr
deBarros2012^†^ [30]	CO	rat	m	10	CO_2_	uo
deFreitas2013 [31]	CO	rat	m	12	CO_2_/He	cr, uo
Demyttenaere2006 [32]	BL	pig	f	15	CO_2_	uo, rbf
Demyttenaere2007^#^ [33]	BL	pig	f	15	CO_2_	uo, rbf
French1951^§^ [34]	CO	dog	f	10	NR	cr, uo, rbf
Ge2009 [12]	CO	rat	m/f	5/10/15/20/25	N_2_	cr
Gong2011 [35]	BL+CO	rat	m	20	N_2_	cr, uo, rbf
Guler1998 [5]	BL+CO	rabbit	NR	10	CO_2_	cr, uo, rbf
Hashikura1994 [36]	BL	pig	NR	6	CO_2_	rbf
Junghans1997^§^^†^ [37]	BL	pig	NR	8/12/16	CO_2_/Ar/He	rbf
Kacmaz2003 [38]	CO	rat	m/f	20	CO_2_	cr
Ke2012 [39]	BL+CO	pig	f	30	N_2_	cr, uo
Khoury2008^†^ [40]	CO	rat	m	5/8/12/15/18	CO_2_	cr, uo
Kirsch1994^#^ [41]	BL+CO	rat	m	5/10	CO_2_	cr, uo
Lee1999^#^^†^ [42]	CO	rat	m	12–18	CO_2_	cr
Li2015^§^ [43]	CO	rabbit	m	6/9/12/15	CO_2_	cr
Lindberg2003 [44]	BL+CO	pig	m/f	12	CO_2_	uo, rbf
Lindstrom2003a [45]	BL+CO	rat	m	5/10	CO_2_	uo
Lindstrom2003b [46]	CO	rat	m	5/10	CO_2_	uo
London2000^§^ [47]	BL	pig	f	15	CO_2_	rbf
McDougall1996^#^^§^ [1]	CO	pig	f	5/10/15/20	CO_2_/Ar	uo, rbf
Moller2012 [48]	BL+CO	pig	f	25	CO_2_	cr, uo
Naffaa2013 [49]	BL	rat	m	10	CO_2_	rbf
Rosin2002 [50]	BL	pig	m	5	N_2_	rbf
Saracoglu2013^#^ [51]	CO	rat	m	20	air	rbf
Schachtrupp2002 [52]	BL+CO	pig	m	15	CO_2_	cr, uo
Schachtrupp2005^#^ [53]	BL+CO	pig	m	30	CO_2_	cr, uo
Schafer2001 [54]	BL*	rat	m	4	CO_2_	rbf
Sener2003 [55]	CO	rat	m/f	20	CO_2_	cr
Shimazutsu2009 [56]	BL+CO	pig	m/f	15	CO_2_	cr, rbf
Shuto1995 [57]	BL	pig	f	8	CO_2_/He	uo, rbf
Tanaka2002 [58]	BL+CO	rat	m	10	CO_2_	uo
Tsugawa1999 [59]	BL	rat	m	10	CO_2_	cr, rbf
Varshavavskii1967^†^ [60]	BL	mouse, rat, dog	NR	NR	air/O_2_	uo
Wiesenthal2011^#^^§^ [61]	BL	rat	NR	5	CO_2_/MA	rbf
Xu2012^§^^†^ [13]	BL+CO	rat	m/f	5/10/15	CO_2_	cr
Yavuz2001[62]	BL+CO	pig	m/f	5 /15	CO_2_	rbf

^#^author provided additional data;

^§^not included in meta-analysis due to missing data;

^†^not included in meta-analysis due to post-PnP data only;

*with laparotomy;

IAP = intra-abdominal pressure;

m = male; f = female; NR = not reported; CO = separate control group; BL = control measurements taken at baseline in the same animals; CO_2_ = carbon dioxide; He = helium; N_2_ = nitrogen; Ar = argon; O_2_ = oxygen; MA = medical air; uo = urine output; rbf = renal blood flow; cr = serum creatinine. For pressure: / = different experimental groups, with different pressures.

Twenty-three studies reported serum creatinine, four (17%) of which had missing data, *i*.*e*. either the mean, variance and/or number of animals could not be extracted. Similarly, thirty studies reported renal blood flow, of which three (10%) had missing data and twenty-nine studies reported urine output, five (17%) of which had missing data. There studies could therefore not be included in the meta-analysis. We did not identify any eligible studies assessing renal histological damage using the Jablonski damage score, therefore no meta-analysis could be conducted for this outcome.

### Study quality and risk of bias assessment

**Fig 2:**
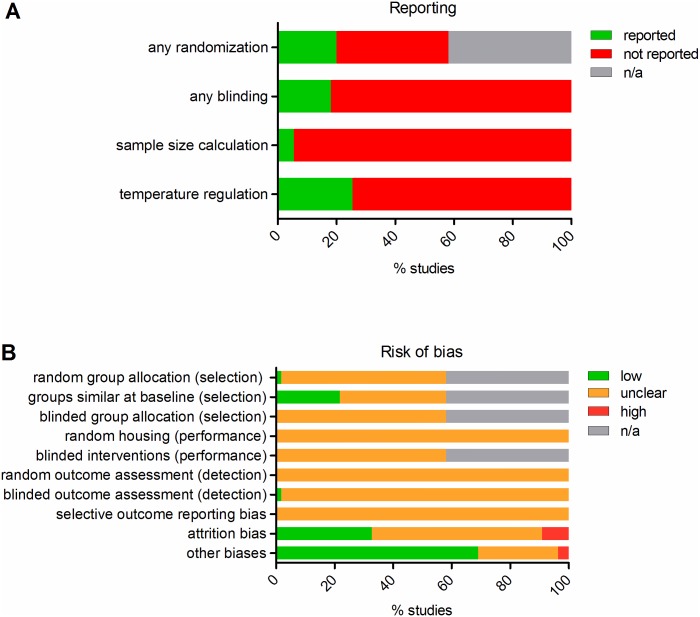
Risk of bias and quality assessment. Poor reporting of key study quality indicators (**A**) resulted in an unclear risk of bias for most types of bias (**B**). In some studies, unexplained drop-outs, and the absence of a Veress needle in the control group, led to a high risk of respectively attrition (item #9) and other biases (item #10). n/a = reporting of randomization (panel A) and items on selection bias (#1–3) and blinding of the intervention (item #5; panel B) were not applicable to Δ baseline studies.

The study quality and risk of bias scores of each individual study are presented in S1 Table. Fig 2 shows the overall results of the study quality and risk of bias assessment for all included studies. Reporting of four key study quality indicators was poor (Fig 2A): out of 55 included studies, eleven studies (20%) mentioned the term “randomisation” in relation to group allocation of the animals. Only one study provided any details on the method of randomisation used. Ten studies (18%) reported blinding during any phase of the experiment, in most cases this concerned blinded outcome assessment of histology. Only three studies (6%) reported using a power calculation to justify the chosen sample size. Fourteen studies (26%) reported that the body temperature of the animals was maintained at a normal physiological level and regulated within a 2°C range, even though differences in body temperature may greatly influence renal damage and other physiological processes.

Because of the poor reporting, the risk of many forms of bias was assessed to be unclear in most studies (Fig 2B). In twelve studies (22%) the baseline characteristics of the animals were described in sufficient detail to assess them as being equal in all experimental groups, resulting in a low risk of selection bias (item 2). In 32 studies (58%) there was an unclear risk of attrition bias, because either the number of drop-outs or the reason for exclusion were not adequately reported. In five studies (9%) there was a high risk of attrition bias since animals appeared to be missing from the analysis, but no explanation was provided. The risk of reporting bias (item 9) was assessed as unclear for all studies, since none of the studies reported the use of a study protocol predefining primary and secondary outcomes. An unclear risk of other biases (item 10) was scored for fifteen studies (27%) in which the experimental procedures (apart from the induction of pneumoperitoneum) appeared to differ between the control and the experimental groups. In most cases it was unclear whether a Veress needle was applied in the control group. A high risk of other biases was scored for four studies (2%) which explicitly reported that no Veress needle was applied in the control group. Reporting of randomization and items on selection bias and blinding of the intervention were not applicable (n/a) to the 23 studies using baseline measurements in the same animals as control data.

Very similar results were obtained when analysing study quality and risk of bias of the studies included in the meta-analysis only (data not shown).

### Meta-analysis of outcomes during pneumoperitoneum

#### Serum creatinine

**Fig 3:**
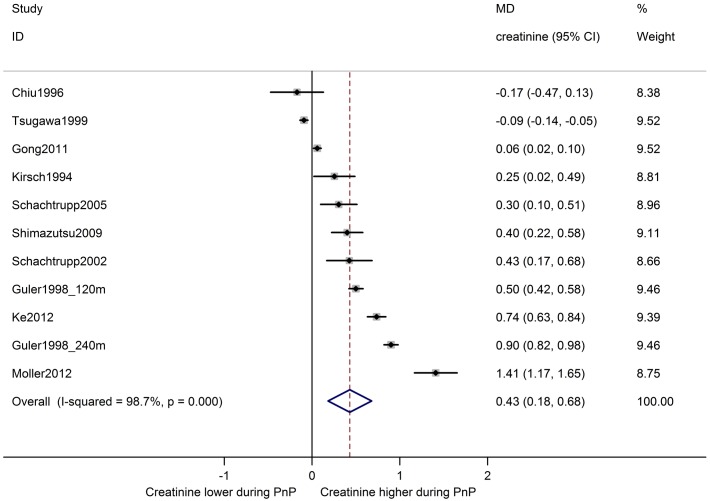
Serum creatinine is increased during pneumoperitoneum compared to baseline. Forest plot of studies comparing serum creatinine during pneumoperitoneum with baseline measurements in the same animals. Effect size is calculated as mean difference (MD) in serum creatinine in mg/dl, and corresponding 95% confidence intervals (95%CI). Weights are from random effects analysis. m = duration of PnP in minutes at time of measurement.

**Fig 4:**
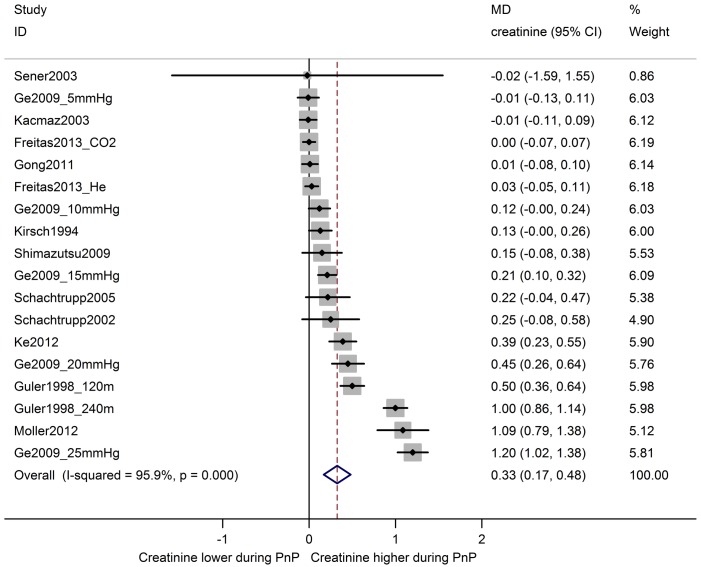
Serum creatinine is increased during pneumoperitoneum compared to controls. Forest plot of studies comparing serum creatinine in animals undergoing pneumoperitoneum with control animals. Effect size is calculated as mean difference (MD) in serum creatinine in mg/dl, and corresponding 95% confidence intervals (95%CI). Weights are from random effects analysis. mmHg = pressure in experimental group; CO2 = insufflation with carbon dioxide; He = insufflation with helium; m = duration in minutes of PnP at time of measurement.

In eleven comparisons from ten studies, serum creatinine levels during pneumoperitoneum were compared with baseline levels in the same animals (Fig 3). Overall, serum creatinine was increased during pneumoperitoneum by MD 0.43 [0.18, 0.68] mg/dl. Between-study heterogeneity was high (I^2^ 99%). Similarly, an overall increase in serum creatinine of MD 0.33 [0.17, 0.48] mg/dl was observed in eighteen comparisons from twelve studies comparing animals undergoing pneumoperitoneum with a separate control group (Fig 4). Between-study heterogeneity was high (I^2^ 96%).

The results of the subgroup analyses of serum creatinine are shown in Table 2. A significant difference in effect between species was observed in Δ baseline studies, where pneumoperitoneum increased serum creatinine in pigs, but not in rats (ΔMD 0.47[0.14, 0.80]). In studies with a separate control group, the same trend was observed, but the confidence intervals of the subgroup effect estimates overlapped. For both Δ baseline studies and control group studies, no effect of pneumoperitoneum was observed in studies using male animals, whereas serum creatinine was increased during pneumoperitoneum in females and mixed groups. However, the confidence intervals of the subgroups overlapped. Serum creatinine was increased in the high pressure subgroup in Δ baseline studies, but not in the medium pressure subgroup (low pressure not analyzed). In control group studies, serum creatinine was increased in the high and medium pressure subgroups, but no effect was observed in the low pressure subgroup. This indicates that serum creatinine may rise with increasing IAP levels, however, the confidence intervals of all subgroups overlapped. The type of gas used for insufflation did not affect the effect of pneumoperitoneum on serum creatinine. For Δ baseline studies, there was no effect on serum creatinine in studies where pneumoperitoneum duration was 61–120 minutes, while serum creatinine was increased when the duration was 121–240 or >240 minutes. For control group studies, there was no effect in the subgroups with 61–120 or 121–240 minutes of pneumoperitoneum, but serum creatinine was increased when the duration was 31–60 or >240 minutes. However, all confidence intervals overlapped between subgroups.

**Table 2:** Subgroup analysis serum creatinine.

	Δ baseline studies					Control group studies				
subgroup	n	MD	95% CI	P-value	I^2^%	n	MD	95% CI	P-value	I^2^%
overall	11	0.43	[0.18, 0.68]	0.001	99	18	0.33	[0.17, 0.48]	0.000	96
*species*										
dog	0	NA	NA	NA	NA	0	NA	NA	NA	NA
pig	6	0.53	[0.33, 0.73]^†^	0.000	94	5	0.41	[0.16, 0.66]	0.001	74
rabbit	2	NA	NA	NA	NA	2	NA	NA	NA	NA
rat	3	0.06	[-0.20, 0.32]^†^	0.654	93	11	0.20	[0.04, 0.36]	0.015	76
*sex*										
female	3	0.68	[0.29, 1.07]	0.001	97	2	NA	NA	NA	NA
male	4	0.25	[-0.08, 0.59]	0.134	79	6	0.09	[-0.12, 0.31]	0.384	26
mixed	2	NA	NA	NA	NA	8	0.29	[0.10, 0.48]	0.003	96
NR	2	NA	NA	NA	NA	2	NA	NA	NA	NA
*pnp pressure*										
low	0	NA	NA	NA	NA	1	-0.01	[-0.69, 0.67]	0.977	0
medium	7	0.32	[-0.06, 0.70]	0.097	99	9	0.26	[0.03, 0.50]	0.024	96
high	4	0.62	[0.12, 1.12]	0.015	99	8	0.45	[0.19, 0.71]	0.001	97
*gas type*										
CO_2_	9	0.44	[0.10, 0.77]	0.011	99	10	0.35	[0.13, 0.58]	0.002	96
other	2	NA	NA	NA	NA	8	0.29	[0.06, 0.53]	0.016	96
*pnp duration*										
1–30 min	2	NA	NA	NA	NA	0	NA	NA	NA	NA
31–60 min	2	NA	NA	NA	NA	9	0.86	[0.22, 1.49]	0.008	85
61–120 min	4	0.66	[-0.17, 1.49]	0.120	72	6	0.74	[-0.07, 1.56]	0.075	70
121–240 min	5	1.21	[0.43, 1.99]	0.002	81	5	0.59	[-0.30, 1.47]	0.195	79
>240 min	4	1.61	[0.82, 2.40]	0.000	87	4	1.17	[0.28, 2.06]	0.010	71

After correction for multiple testing of 4 variables, P<0.012 was considered significant for Δ baseline studies. After correction for multiple testing of 5 variables, P<0.010 was considered significant for control group studies.

^†^significant difference between these two subgroups;

n = number of comparisons in analysis; CI = confidence interval; pnp = pneumoperitoneum; NA = not analyzed due to insufficient data

Heterogeneity was high in the overall analysis for both Δ baseline studies and control group studies, and there was high residual heterogeneity in nearly all subgroups, except for control group studies conducted in male animals. None of the analyzed subgroup variables accounted for a significant proportion of heterogeneity.

#### Renal Blood flow

**Fig 5:**
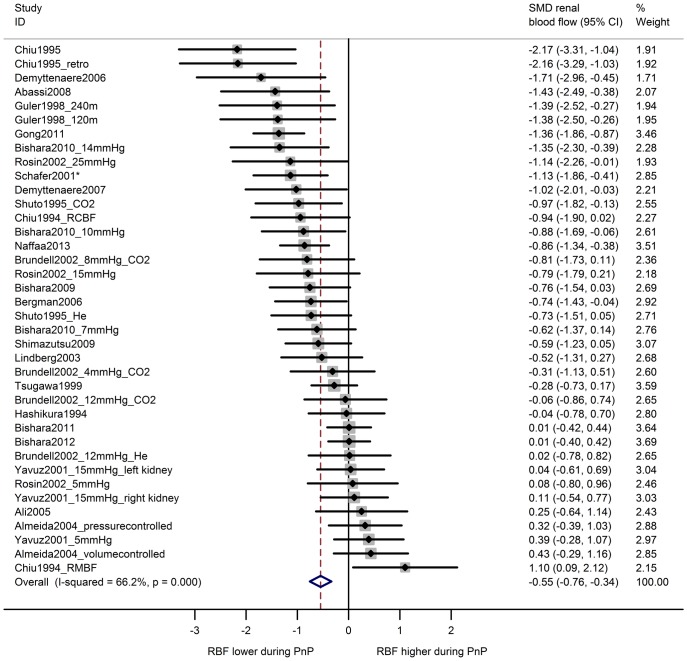
Renal blood flow is decreased during pneumoperitoneum compared to baseline. Forest plot of studies comparing renal blood flow during pneumoperitoneum with baseline measurements in the same animals. Effect size is calculated as standardized mean difference (SMD) in renal blood flow, and corresponding 95% confidence intervals (95%CI). Weights are from random effects analysis. *baseline measurements during laparotomy; retro = retroperitoneum applied; m = duration in minutes of PnP at time of measurement; mmHg = pressure in experimental group; CO2 = insufflation with carbon dioxide; He = insufflation with helium; RCBF = renal cortical blood flow; RMBF = renal medullary blood flow.

**Fig 6:**
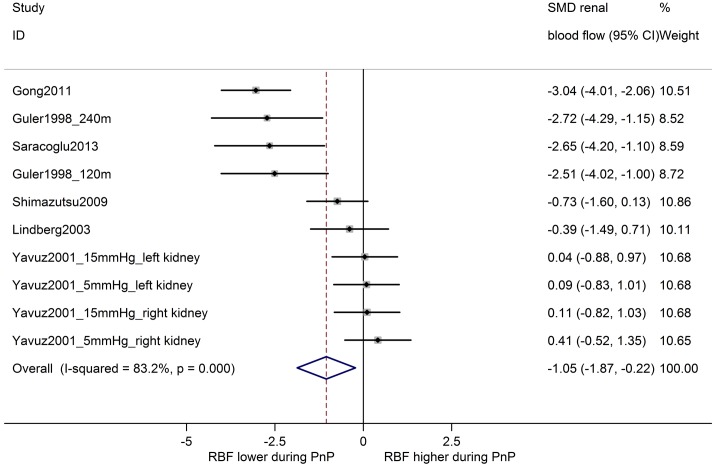
Renal blood flow is decreased during pneumoperitoneum compared to controls. Forest plot of studies comparing renal blood flow in animals undergoing pneumoperitoneum with control animals. Effect size is calculated as standardized mean difference (SMD) in renal blood flow, and corresponding 95% confidence intervals (95%CI). Weights are from random effects analysis. m = duration in minutes of PnP at time of measurement; mmHg = pressure in experimental group.

In 38 comparisons from 24 studies, renal blood flow during pneumoperitoneum was compared with baseline levels in the same animals (Fig 5). Overall, renal blood flow was decreased during pneumoperitoneum (SMD -0.55 [-0.76, -0.34] and between-study heterogeneity was moderate to high (I^2^ 66%). Similarly, an overall decrease in renal blood flow was observed for ten comparisons from six studies comparing animals undergoing pneumoperitoneum with a separate control group ((SMD -1.05 [0–1.87, -0.22]); Fig 6). Between-study heterogeneity was high (I^2^ 83%).

The results of the subgroup analyses of renal blood flow are shown in Table 3. Data were insufficient to assess the effect of species, sex, IAP level and gas type in control group studies. In control group studies, renal blood flow was decreased in the subgroups of studies with a pneumoperitoneum duration 121–240 minutes, but there was no effect in the subgroup with a duration of 1–30 minutes. However, the confidence intervals of these subgroups overlapped.

**Table 3:** Subgroup analysis renal blood flow.

	Δ baseline studies					Control group studies				
subgroup	n	SMD	95% CI	P-value	I^2^%	n	SMD	95% CI	P-value	I^2^%
overall	29	-0.47	[-0.68, -0.26]	0.000	60	10	-1.05	[-1.87, -0.22]	0.013	83
*species*										
dog	2	NA	NA	NA	NA	0	NA	NA	NA	NA
pig	17	-0.44	[-0.71, -0.17]	0.002	35	6	-0.08	[-0.46, 0.30]	0.678	0
rabbit	0	NA	NA	NA	NA	2	NA	NA	NA	NA
rat	10	-0.66	[-0.97, -0.34]	0.000	73	2	NA	NA	NA	NA
*sex*										
female	9	-0.64	[-1.01, -0.26]	0.001	15	0	NA	NA	NA	NA
male	13	-0.64	[-0.92, -0.36] ^†^	0.000	67	2	NA	NA	NA	NA
mixed	7	0.03	[-0.35, 0.41] ^†^	0.883	30	6	-0.08	[-0.46, 0.30]	0.678	0
NR	0	NA	NA	NA	NA	2	NA	NA	NA	NA
*pnp pressure*										
low	10	-0.31	[-0.62, 0.00]	0.053	41	2	NA	NA	NA	NA
medium	17	-0.46	[-0.70, -0.21]	0.000	54	6	-0.82	[-1.54, -0.11]	0.024	71
high	2	NA	NA	NA	NA	2	NA	NA	NA	NA
*gas type*										
CO_2_	23	-0.42	[-0.64, -0.19]	0.000	55	8	-0.54	[-1.17, 0.09]	0.096	69
other	6	-0.69	[-1.15, -0.22]	0.004	62	2	NA	NA	NA	NA
*pnp duration*										
1–30 min	28	-0.59	[-0.81, -0.37]	0.000	47	5	0.01	[-0.88, 0.90]	0.978	0
31–60 min	14	-0.65	[-0.96, -0.35]	0.000	64	1	NA	NA	NA	NA
61–120 min	4	-1.11	[-1.74, -0.48]	0.001	62	2	NA	NA	NA	NA
121–240 min	4	-0.83	[-1.39, -0.27]	0.004	70	4	-1.57	[-2.61, -0.54]	0.003	86
>240 min	0	NA	NA	NA	NA	0	NA	NA	NA	NA

After correction for multiple testing of 5 variables, P<0.010 was considered significant for Δ baseline studies. For control group studies only 1 variable was tested, therefore P<0.05 was considered significant;

^†^significant difference between these two subgroups;

n = number of comparisons in analysis; CI = confidence interval; pnp = pneumoperitoneum; NA = not analyzed due to insufficient data.

In Δ baseline studies, pneumoperitoneum caused a decrease in renal blood flow in both species and no difference between the species was observed. When assessing the influence of sex, a decrease in renal blood flow was observed in studies using male and female animals, but not in studies using mixed sex groups. The difference in effect was significant between males and mixed sex animals (ΔSMD 0.67 [020, 1.14]). Subgroup analysis on the level of IAP in Δ baseline studies showed a decrease in renal blood flow in both the low and medium pressure group, with no significant difference between the groups. The type of gas used for insufflation and the duration of pneumoperitoneum also did not influence the effect of pneumoperitoneum on renal blood flow.

For Δ baseline studies, heterogeneity was moderate in the overall analysis. None of the analyzed subgroup variables accounted for a significant proportion of heterogeneity. Heterogeneity was reduced in the subgroups of females and mixed sexes, but this did not reach statistical significance. In the other subgroups, residual heterogeneity was moderate to high. Similarly, for control group studies subgroup variables could not account for significant proportion of the high heterogeneity found in the overall analysis. Residual heterogeneity in subgroups either remained moderate to high, or was reduced to 0.

#### Urine output

**Fig 7:**
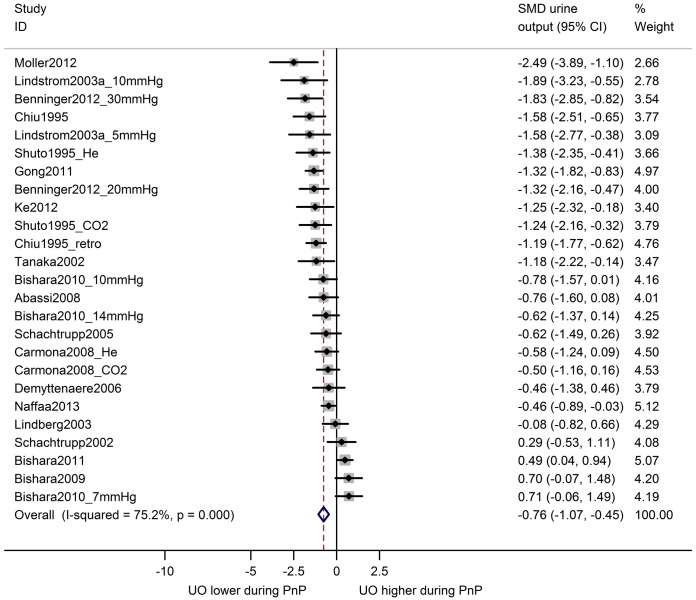
Urine output is decreased during pneumoperitoneum compared to baseline. Forest plot of studies comparing urine output during pneumoperitoneum with baseline measurements in the same animals. Effect size is calculated as standardized mean difference (SMD) in urine output, and corresponding 95% confidence intervals (95%CI). Weights are from random effects analysis. mmHg = pressure in experimental group; m = duration in minutes of PnP at time of measurement; He = insufflation with helium; CO2 = insufflation with carbon dioxide; retro = retroperitoneum applied.

**Fig 8:**
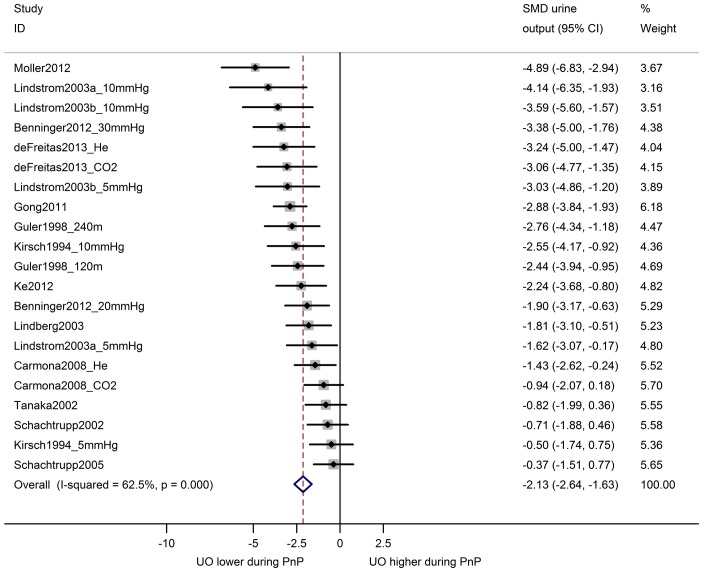
Urine output is decreased during pneumoperitoneum compared to controls. Forest plot of studies comparing urine output in animals undergoing pneumoperitoneum with control animals. Effect size is calculated as standardized mean difference (SMD) in urine output, and corresponding 95% confidence intervals (95%CI). Weights are from random effects analysis. mmHg = pressure in experimental group; He = insufflation with helium; CO2 = insufflation with carbon dioxide; m = duration in minutes of PnP at time of measurement.

In 25 comparisons from 18 studies, urine output during pneumoperitoneum was compared with baseline levels in the same animals (Fig 7). Overall, urine output was decreased during pneumoperitoneum (SMD -0.76 [-1.07, -0.45]) and between-study heterogeneity was high (I^2^ 75%). Similarly, an overall decrease in renal blood flow was observed for 21 comparisons from 14 studies comparing animals undergoing pneumoperitoneum with a separate control group (SMD -2.13 [-2.64, -1.63]; Fig 8). Between-study heterogeneity was moderate (I^2^ 63%).

The results of the subgroup analyses of urine output are shown in Table 4. In both Δ baseline studies and control group studies, the variables species and sex did not influence the effect of pneumoperitoneum on outcome, since urine output was decreased during pneumoperitoneum in all sexes and species, and all confidence intervals overlapped. Subgroup analysis on the level of IAP in Δ baseline studies showed a decrease in renal blood flow in the medium and high pressure groups. No effect was observed in the low pressure group, and its effect estimate differed significantly from the high pressure group (ΔSMD 1.18 [0.41, 1.95]). This difference was not observed in control group studies, where urine output was equally reduced in all pressure groups. The type of gas used for insufflation and the duration of pneumoperitoneum did not influence the effect on renal blood flow in either Δ baseline studies or control group studies.

**Table 4:** Subgroup analysis urine output.

	Δ baseline studies					Control group studies				
subgroup	n	SMD	95% CI	P-value	I^2^%	n	SMD	95% CI	P-value	I^2^%
overall	25	-0.76	[-1.07, -0.45]	0.000	75	21	-2.13	[-2.64, -1.63]	0.000	63
*species*										
dog	2	NA	NA	NA	NA	2	NA	NA	NA	NA
pig	12	-1.03	[-1.49, -0.58]	0.000	61	7	-2.00	[-2.88, -1.12]	0.000	74
rabbit	0	NA	NA	NA	NA	2	NA	NA	NA	NA
rat	11	-0.52	[-0.98, -0.06]	0.026	82	10	-2.39	[-3.16, -1.62]	0.000	61
*sex*										
female	5	-1.29	[-2.03, -0.55]	0.001	32	2	NA	NA	NA	NA
male	15	-0.60	[-1.00, -0.20]	0.003	80	14	-2.10	[-2.75, -1.46]	0.000	67
mixed	1	NA	NA	NA	NA	1	NA	NA	NA	NA
NR	4	-0.94	[-1.68, -0.20]	0.012	44	4	-1.82	[-2.99, -0.66]	0.002	35
*pnp pressure*										
low	6	-0.21	[-0.74, 0.32] ^†^	0.443	84	3	-1.59	[-2.97, -0.20]	0.025	61
medium	13	-0.70	[-1.05, -0.34]	0.000	41	12	-2.11	[-2.80, -1.42]	0.000	53
high	6	-1.39	[-1.94, -0.83] ^†^	0.000	19	6	-2.45	[-3.40, -1.50]	0.000	76
*gas type*										
CO_2_	21	-0.68	[-1.02, -0.35]	0.000	76	17	-2.06	[-2.63, -1.49]	0.000	65
other	4	-1.12	[-1.86, -0.37]	0.003	15	4	-2.41	[-3.53, -1.29]	0.000	32
*pnp duration*										
1–30 min	12	-0.49	[-0.88, -0.09]	0.016	62	5	-1.43	[-2.40, -0.46]	0.004	0
31–60 min	19	-0.54	[-0.86, -0.23]	0.001	68	13	-1.33	[-1.94, -0.72]	0.000	54
61–120 min	11	-0.75	[-1.17, -0.34]	0.000	52	16	-1.54	[-2.10, -0.98]	0.000	70
121–240 min	8	-0.76	[-1.25, -0.28]	0.002	68	11	-1.54	[-2.20, -0.88]	0.000	73
>240 min	6	-0.83	[-1.44, -0.22]	0.007	80	6	-1.69	[-2.61, -0.77]	0.000	79

After correction for multiple testing of 5 variables, P<0.010 was considered significant for both Δ baseline studies and control group studies;

^†^significant difference between these two subgroups;

n = number of comparisons in analysis; CI = confidence interval; pnp = pneumoperitoneum; NA = not analyzed due to insufficient data

Heterogeneity was moderate to high in both overall analyses, and remained moderate to high in the majority of subgroups. None of the subgroup variables accounted for a significant proportion of heterogeneity (P<0.05 for subgroup variables).

### Descriptive summary of outcomes measured after pneumoperitoneum

Four studies [18, 19, 40, 42] measured serum creatinine 24 hours or longer after pneumoperitoneum, one study [40] measured urine output after 24 hours and no studies measured renal blood flow at a long-term time-point. In rabbits, Bayar et al. found no difference in serum creatinine compared to controls, 24 hours after a 90-minute, 15mmHg pneumoperitoneum in rats. Bedushi et al. found no difference in serum creatinine on day 7 between rats undergoing a 2 hour, 15mmHg pneumoperitoneum and sham controls. Similarly, Lee et al. reported no difference in serum creatinine *versus* controls three months after a 1 or 5 hour 15mmHg pneumoperitoneum in rats. Khoury et al. observed an increase of serum creatinine with increasing pressure levels (5, 12 and 18 mmHg), as analyzed by linear regression. The latter study found no differences between 30 and 60 minutes of duration for each pressure level.

For urine output, Khoury et al. reported an increase 24 hours after a 60 (but not 30) minute pneumoperitoneum of 18mmHg. For lower pressures, no difference in urine output after 24 hours was observed.

In summary, most of these studies used a moderate pressure level (15mmHg) and found no long-term effects of pneumoperitoneum on renal function. One study found evidence for long-term effects after slightly higher pressure levels (18 mmHg), depending on pneumoperitoneum duration.

### Publication bias

The analyses of serum creatinine data contained too few studies to reliably assess funnel plot asymmetry (twelve control group studies, ten Δ baseline studies).

### Sensitivity analysis

Redefining the pre-specified cut-off points for the subgroups of IAP level and IAP duration did not significantly alter the results of the meta-analysis for any of the outcome measures (data not shown). Omitting the data from Schaeffer 2012 (baseline under laparotomy) did not alter the analysis results (data not shown). We re-analyzed the data after pooling the comparisons from control group studies and Δ baseline studies, removing the Δ baseline data in case of duplicates. The results did not differ significantly from the outcome of the original, separate analyses, although heterogeneity tended to increase when pooling the different study designs (serum creatinine: 20 comparisons, MD 0.28 [0.14, 0.42], I^2^ 96%; RBF: 40 comparisons, SMD -0.65 [-0.89, -0.40], I^2^ 72%; UO: 33 comparisons, SMD -1.45 [-1.87, -1.04], I^2^ 82%). We therefore feel that our decision to analyze data from control group studies and Δ baseline studies separately is justified.

## Discussion

### Main findings and clinical implications

Here we report the first systematic review and meta-analysis of current literature reporting the effect of pneumoperitoneum on renal function in animal models. We performed meta-analyses of the outcome measures serum creatinine, urine output and renal blood flow, and found that detrimental effects on renal function during pneumoperitoneum were reflected by all three outcome measures. These effects were consistent both in studies using a separate control group and in Δ baseline studies, and robust in sensitivity analyses. In daily clinical practice, many laparoscopic surgical procedures are performed under a 12–14 mmHg pneumoperitoneum, which is generally considered to be standard pressure. In specific procedures, *e*.*g*. in gynaecology, even higher pressures may be used. The mean pressure level in our overall analyses was 11–17 mmHg (median 10–15). Thus, our findings are in line with the results of clinical trials reporting adverse effects of (standard pressure) pneumoperitoneum on renal function (*e*.*g*. [63–65]). However, we found that preclinical evidence on the long-term effects of pneumoperitoneum on renal function is scarce. The four studies measuring outcomes ≥24 hours after a 15mmHg pneumoperitoneum suggest that serum creatinine normalises within days or weeks, but this may not be the case for higher pressures, or other renal outcomes.

We performed pre-specified subgroup analyses to explore between-study heterogeneity and to identify factors modifying the effect of pneumoperitoneum on renal function. Regarding population-related characteristics, an effect of species was observed for serum creatinine in Δ baseline studies, for which an increase was found in pigs, but not in rats. However, these results were not observed in control group studies, and was not present for urine output (data for renal blood flow were insufficient). The subgroups for sex were often very small, and the effect of sex on pneumoperitoneum was not consistent between outcome measures. As such, the current body of evidence is insufficient to reliably conclude that either species or sex influence peumoperitoneum-induced renal damage.

Regarding characteristics of the pneumoperitoneum, subgroup analysis of pressure level indicated that the effect of pneumoperitoneum on urine output worsened as IAP level increased. In patients, lowering the IAP pressure during pneumoperitoneum (generally to 6–10 mmHg) can reduce postoperative pain, and may reduce the risk of internal organ dysfunction. International guidelines on the use of pneumoperitoneum in clinical practice therefore recommend the use of the lowest possible pressure still allowing adequate exposure of the operative field [66]. Our findings are in line with two clinical trials comparing standard *versus* low-pressure pneumoperitoneum, which reported that lowering the pressure improved short-term urine output, but found no effect on serum creatinine [67, 68]. Due to insufficient data and high between-study heterogeneity, we were not able to assess whether, as in the clinical trials, these effects in animals are reversible.

In clinical practice, CO_2_ is the gas most widely employed for insufflation, since it is noncombustible, relatively inexpensive and highly soluble in blood, thereby minimizing the risk of embolism. We did not identify a significant difference between subgroups of studies using CO_2_ versus those other types of gas. This is favorable in light of the advantages of CO_2_ in terms of *i*.*a*. availability and combustibility, and the non-renal side effects reported for helium pneumoperitoneum [69]. Although the evidence base is small and of low quality, there presently are no indications that there is an advantage of using any other type of gas when CO_2_ is available.

In some of our analyses we observed a trend towards worsening of renal function as the duration of pneumoperitoneum increased, but this effect was not consistent over all outcome measures and study designs. As a consequence no reliable conclusions regarding the effect of the duration of pneumoperitoneum on renal function could be drawn.

Importantly, the observed changes in renal function and perfusion may reversible and of little importance in generally healthy patients, but may also have significant impact in patients with pre-existing compromised renal function. This warrants future research in animal models with relevant co-morbidities, such as chronic kidney disease. Of note, our search identified 9 studies using animals with any type of co-morbidity, only one of which was directly related to the kidney (renal cancer; the other 8 concerned pancreatitis, peritonitis, non-renal carcinomas and gastric ulcers). We did not identify any studies reporting data histological damage using the Jablonski score for renal damage, and were therefore unable to assess whether the observed changes in renal function and perfusion were accompanied by histopathological changes.

In addition to direct effects of pneumoperitoneum on the kidney and renal vasculature, renal function may also be affected by cardiac, pulmonary and neurohormonal consequences of pneumoperitoneum. Although it was beyond the scope of this review to assess the involvement of these systems, we feel that this would be an important question to address in the future.

### Limitations of this review

#### Methodological quality and risk of bias

Adequate reporting of methodological details is crucial to determine the risk of bias in primary studies, and to assess the quality of a body of evidence. Our quality assessment shows that key methodological aspects of the primary studies were often not reported in sufficient detail, or not reported at all. As a result, the risk of bias in most studies is unclear. Although this does not necessarily mean that the studies were in fact methodologically impaired (which is why the studies were not excluded from the review), our findings are reason for concern, since insufficient reporting of research methodology in preclinical studies is often associated with an overestimation of treatment effects [70]. Consequently, the results of the included studies may have been affected by bias, which may have influenced the outcome of our meta-analysis.

Of note, only three studies reported a power calculation justifying the chosen group sizes, while the number of animals per group was low in many studies (median 8, range 6–30, not taking into account multiple comparisons with a single control group). As a result, it is unclear whether or not the individual studies were adequately powered, which is unfortunate since the risk of finding false positive results is increased in underpowered studies. Thus, we cannot exclude the possibility of an effect of underpowering on our meta-analysis.

#### Between-study heterogeneity

The number of studies in many of the (subgroup) analyses is low, and the extracted data are highly heterogeneous. Data were insufficient to perform some of the planned analyses, *e*.*g*. on the long-term effects of pneumoperitoneum, and a number of subgroup analysis. We have accounted for anticipated heterogeneity by performing random rather than fixed effects meta-analysis. Furthermore, we aimed to identify variables explaining heterogeneity, but found that a high proportion of residual, unexplained, heterogeneity remained after subgroup analysis. We hypothesise that this heterogeneity arises from between-study differences in design and quality, but were unable to test this hypothesis due to insufficient reporting and limited data. To increase our confidence in the results in spite of the residual heterogeneity, we conducted multiple sensitivity analyses, which indicate that our findings are robust.

#### Publication bias

There was insufficient data to perform reliable assessment of publication bias, and the risk of publication bias in this body of evidence is therefore unclear.

## Conclusions and recommendations

The body of preclinical evidence on the effects of pneumoperitoneum on renal function and perfusion is highly heterogeneous and of questionable quality. Overall, there appear to be robust adverse effects of pneumoperitoneum on serum creatinine, renal blood flow and urine output, but the reliability and clinical relevance of these findings remain uncertain, as we are presently unable to assess the long-term effects of pneumoperitoneum. This review emphasizes the need for rigorous reporting of preclinical research methodology (including measures to reduce bias), which is of vital importance for the correct interpretation and translation of preclinical data.

## Supporting Information

S1 File Systematic review protocol. (PDF)

S2 File Full search strategy. (PDF)

S3 File PRISMA checklist. (PDF)

S1 Folder CMA meta-analysis files. (ZIP)

S1 Table Individual quality assessment and risk of bias scores. (PDF)
