# Supplementary material for: Renal Perfusion and Function during Pneumoperitoneum: A Systematic Review and Meta-Analysis of Animal Studies
Source: PLoS One. 2016 Sep 22;11(9):e0163419. doi: 10.1371/journal.pone.0163419 (PMC5033590; doi:10.1371/journal.pone.0163419)
Supplement: S1 File — (PDF) [file pone.0163419.s001.pdf]

SYSTEMATIC REVIEW PROTOCOL FOR ANIMAL INTERVENTION STUDIES

FORMAT BY SYRCLE (WWW.SYRCLE.NL)
VERSION 2.0 (DECEMBER 2014)

Item #	Section/Subsection/Item	Description	Check for approval
	A. General		
1.	Title of the review	Influence of pneumoperitoneum on renal function: a systematic review and meta-analysis of animal studies	X
2.	Authors (names, affiliations, contributions)	Moira H.D. Bruintjes1<br>Kimberley E. Wever[1,2]<br>Carlijn R. Hooijmans2<br>Michiel C. Warlé1<br>Departments of [1]Surgery, [2] SYstematic Review Centre for Laboratory animal Experimentation (SYRCLE), Radboud UMC, Nijmegen, The Netherlands	X
3.	Other contributors (names, affiliations, contributions)	Alice Tillema, Medical Library, Radboud UMC: search strategy design	X
4.	Contact person + e-mail address	Moira Bruintjes: moirabruintjes@gmail.com	X
5.	Funding sources/sponsors	Funded by departments of surgery and SYRCLE	X
6.	Conflicts of interest	No conflicts of interests	X
7.	Date and location of protocol registration	-	
8.	Registration number (if applicable)	-	
9.	Stage of review at time of registration	-	
	B. Objectives		
	Background		
10.	What is already known about this disease/ model/ intervention? Why is it important to do this review?	Raised IAP during laparoscopic procedures can affect several homeostatic systems, causing alterations in cardiovascular, pulmonary and renal physiology. Reported renal effects include oliguria or anuria, decreased renal blood flow, decreased glomerular filtration rate, decreased reabsorption of glucose, increased reabsorption of water, increased renal venous pressure and proteinuria. There is a huge variation in the study designs and animal models used (*e.g.* various animal species, magnitudes of IAP and outcome measures), which makes it difficult to compare them directly.<br>We will provide an overview of all animal studies on the effects of pneumoperitoneum on renal function and an overview of the methodological quality of all these studies (evaluated through a Risk of Bias assessment).<br>We will perform a meta-analysis of all studies to answer the question if pneumoperitoneum has significant adverse effects on renal functioning. Furthermore we will investigate the different factors affecting the renal injury after pneumoperitoneum (*e.g.* duration of pneumoperitoneum, level of pressure, type of gas, age and gender).	X

	Research question		
11.	Specify the disease / health problem of interest	Renal damage due to pneumoperitoneum	X
12.	Specify the population /species studied	All animal species	X
13.	Specify the intervention/exposure	Pneumoperitoneum	X
14.	Specify the control population	No/ low pressure pneumoperitoneum , including baseline recordings, no intervention, sham operation or laparotomy	X
15.	Specify the outcome measures	Serum creatinine, diuresis, renal blood flow, renal histology	X
16.	State your research question (based on items 11-15)	Does pneumoperitoneum cause renal damage in animals?	X
	C. Methods		
	Search and study identification		
17.	Identify literature databases to search (*e.g.* Pubmed, Embase, Web of science)	X MEDLINE via PubMed ☐Web of Science<br>☐SCOPUS X EMBASE<br>☐Other, namely:<br>☐Specific journal(s), namely:	X
18.	Define electronic search strategies (*e.g.* use the step by step search guide [1] and animal search filters [2, 3])	Supplementary file containing search strategy: "Search strategy pneumo animal_KW.doc" (available at request of contact person)	X
19.	Identify other sources for study identification	X Reference lists of included studies ☐Books<br>X Reference lists of relevant reviews<br>☐Conference proceedings, namely:<br>☐Contacting authors/ organisations, namely:<br>☐Other, namely:	X
20.	Define search strategy for these other sources	Screening the reference lists for relevant titles and screening the abstracts of these relevant titles	X
	Study selection		
21.	Define screening phases (*e.g.* pre-screening based on title/abstract, full text screening, both)	1) screening based on title and abstract<br>2) full-text screening of the eligible articles	X
22.	Specify (a) the number of reviewers per screening phase and (b) how discrepancies will be resolved	Each phase: 2 independent observers per article. One observer will screen all articles (MB), KW and CH will each screen half of the articles. Differences will be solved through discussion or by consulting a fourth investigator.	X
	Define all inclusion and exclusion criteria based on:		
23.	Type of study (design)	Inclusion criteria: comparison of high versus no/low intra-abdominal pressure<br>Exclusion criteria: No high versus no/low intra-abdominal pressure. Co-interventions (*e.g* .nephrectomy, co-medication)	X

24.	Type of animals/ population (*e.g.* age, gender, disease model)	Inclusion criteria: healthy animals of all species, genders and ages<br>Exclusion criteria: co-morbidity, knock-out animals, *ex-vivo, in vitro, in silico*	X
25.	Type of intervention (*e.g.* dosage, timing, frequency)	Inclusion criteria: pneumoperitoneum by insufflations with all types of gas<br>Exclusion criteria: other methods to increase intra-abdominal pressure, *e.g.* fluid or balloon inflation	X
26.	Outcome measures	Inclusion criteria: serum creatinine, diuresis, renal blood flow, renal histology assessed with Jablonski's scale for renal histological damage<br>Exclusion criteria: no relevant outcome measure	X
27.	Language restrictions	Inclusion criteria: all languages<br>Exclusion criteria: none	X
28.	Publication date restrictions	Inclusion criteria: all publication dates<br>Exclusion criteria: none	X
29.	Other	NA	
30.	Sort and prioritize your exclusion criteria per selection phase	Selection phase tiab screening:<br>1. Review<br>2. No full paper (abstract, comment)<br>3. Data published in duplicate<br>4. Human<br>5. Not *in vivo* (*e.g. ex vivo*/*in vitro*/*in sillico*)<br>6. No comparison high *versus* no/low pressure<br>7. Fluid or balloon inflation<br>8. Co-intervention or co-medication<br>9. Co-morbidity<br>10. No relevant outcome measure<br><br>**Additional** criteria for full text screening:<br>11. Co-intervention, co-morbidity or co-medication<br>12. No relevant outcome measure	X
	Study characteristics to be extracted (for assessment of external validity, reporting quality)		
31.	Study ID (*e.g.* authors, year)	Authors, title, year, language, contact author e-mail	X
32.	Study design characteristics (*e.g.* experimental groups, number of animals)	Number of animals in experimental and control groups, presence of control group, body temperature during intervention, power calculation reported	X
33.	Animal model characteristics (*e.g.* species, gender, disease induction)	Animal species, strain, age, weight, gender	X
34.	Intervention characteristics (*e.g.* intervention, timing, duration)	Type of control intervention, pressure in control and experimental group, type of gas used, pressure duration, timing measurements	X
35.	Outcome measures	Serum creatinine, diuresis, renal histology assessed with Jablonski's scale for renal histological damage, and renal blood flow Y/N, presence of any other outcome measures	X

		(key-words)	
36.	Other (*e.g.* drop-outs)	Number of excluded animals, reason of exclusion	X
	Assessment risk of bias (internal validity) or study quality		
37.	Specify (a) the number of reviewers assessing the risk of bias/study quality in each study and (b) how discrepancies will be resolved	a) 3 reviewers. The criteria will be independently assessed by MB,KW and CH by using collectively predefined assessment criteria. b) discrepancies will be resolved by discussion	X
38.	Define criteria to assess (a) the internal validity of included studies (*e.g.* selection, performance, detection and attrition bias) and/or (b) other study quality measures (*e.g.* reporting quality, power)	☐By use of SYRCLE's Risk of Bias tool [4]<br>**X** By use of SYRCLE's Risk of Bias tool, adapted as follows: Because of poor reporting of essential details in animal studies, we will also include one reporting item: was it stated that the experiment was randomized at any level?<br>☐By use of CAMARADES' study quality checklist, e.g. [5]<br>☐By use of CAMARADES' study quality checklist, adapted as follows:<br>☐Other criteria, namely:	X
	Collection of outcome data		
39.	For each outcome measure, define the type of data to be extracted (*e.g.* continuous/ dichotomous, unit of measurement)	Serum creatinine: continuous, unit: mg/dL, mmol/L. Final unit: mg/dL<br>Diuresis: continuous, unit: mL/min, mL/kg, µL/min/g, µL/min/g kidney weight.<br>Renal blood flow: continuous, unit: mL/min, mL/min/100mg (kidney weight), cc/min, U/min<br>Renal histology: assessed with Jablonski's scale for renal histological damage, scale 0-5.	X
40.	Methods for data extraction/retrieval (*e.g.* first extraction from graphs using a digital screen ruler, then contacting authors)	Extraction from text and tables.<br>Contacting authors by e-mail.<br>Extraction from graphs using digital image analysis software (ImageJ; http://rsbweb.nih.gov/ij/) by two independent reviewers.	X
41.	Specify (a) the number of reviewers extracting data and (b) how discrepancies will be resolved	a) Two reviewers (MB and KW) will extract all data. b) discrepancies will be resolved by discussion	X
	Data analysis/synthesis		
42.	Specify (per outcome measure) how you are planning to combine/compare the data (*e.g.* descriptive summary, meta-analysis)	Meta-analysis with subgroup analysis and sensitivity analysis for all outcome measures	X
43.	Specify (per outcome measure) how it will be decided whether a meta-analysis will be performed	A minimum of 5 articles per outcome measure is required. No restrictions in terms of heterogeneity will be applied, instead, sources of heterogeneity will be investigated through sensitivity and subgroup analysis.	X
	If a meta-analysis seems feasible/sensible, specify (for each outcome measure):		
44.	The effect measure to be used (*e.g.* mean difference, standardized mean difference, risk ratio, odds ratio)	Serum creatinine: MD (all data converted to mg/dl)<br>Diuresis: SMD or MD (depending on reported units and whether these can be converted to a single unit)<br>Renal blood flow: SMD	X

		Renal histology: MD	
45.	The statistical model of analysis (*e.g.* random or fixed effects model)	Random effects model	X
46.	The statistical methods to assess heterogeneity (*e.g.* I^2, Q)	I^2	X
47.	Which study characteristics will be examined as potential source of heterogeneity (subgroup analysis)	Animal species (mouse *vs.* rat *vs.* pig etc)<br>Gender (male *vs.* female *vs.* mixed)<br>Pressure of pneumoperitoneum (high/ medium/ low)<br>Duration of pneumoperitoneum (short/ medium/ long)<br>Type of gas used (CO_2 *vs.* helium etc.)	X
48.	Any sensitivity analyses you propose to perform	Cut-off points for pressure grouping into high-medium-low<br>Cut-off points for duration grouping into short-medium-long<br>Pooling of baseline-controlled studies with studies using a control group<br>Study quality	X
49.	Other details meta-analysis (*e.g.* correction for multiple testing, correction for multiple use of control group)	If studies report data for a series of time points, we will pool the data of different time points to correct for repeated measurements of dependent variables. We will correct for repeated use of the same control group by dividing the number of animals in the control group by the number of comparisons made.	X
50.	The method for assessment of publication bias	Critical visual inspection of Funnel plots	X

Final approval by (names, affiliations):	Drs. Moira Bruintjes, dept. of Surgery<br>Dr. Kim Wever, dept. of Surgery and SYRCLE<br>Dr. Michiel Warlé, dept. of Surgery<br>Dr. Carlijn Hooijmans , SYRCLE	Date: 26-9-2013
