# Supplementary material for: Renal Perfusion and Function during Pneumoperitoneum: A Systematic Review and Meta-Analysis of Animal Studies
Source: PLoS One. 2016 Sep 22;11(9):e0163419. doi: 10.1371/journal.pone.0163419 (PMC5033590; doi:10.1371/journal.pone.0163419)
Supplement: S1 Table — (PDF) [file pone.0163419.s005.pdf]

S1 Table: Individual risk of bias scores

study	control data type	any randomisation	any blinding	sample size calculation	temperature control	groups similar at baseline (selection)	random group allocation (selection)	blinded group allocation (selection)	random housing (performance)	blinded interventions (performance)	blinded outcome assessment (detection)	random outcome assessment (detection)	attrition bias	selective outcome reporting bias	other biases
Abassi2008 [14]	BL	NA	N	N	N	NA	NA	NA	?	NA	?	?	?	?	L
Ali2005 [15]	BL	NA	N	N	N	NA	NA	NA	?	NA	?	?	L	?	L
Almeida2004 [16]	BL	NA	N	N	N	NA	NA	NA	?	NA	?	?	?	?	?
Avraamidou2012§ [17]	BL+CO	N	N	N	N	?	?	?	?	?	?	?	?	?	?
Bayar2006† [18]	BL+CO	Y	N	N	Y	?	?	?	?	?	?	?	L	?	L
Beduschi1999† [19]	BL+CO	N	N	Y	N	?	?	?	?	?	?	?	H	?	?
Benninger2012 [20]	BL+CO	N	N	N	N	L	?	?	?	?	?	?	?	?	L
Bergman2006 [21]	BL	NA	N	Y	N	NA	NA	NA	?	NA	?	?	H	?	?
Bishara2009 [22]	BL	NA	N	N	N	NA	NA	NA	?	NA	?	?	?	?	L
Bishara2010 [23]	BL	N	N	N	N	L	?	?	?	?	?	?	?	?	L
Bishara2011 [24]	BL	NA	N	N	N	NA	NA	NA	?	NA	?	?	?	?	L
Bishara2012 [25]	BL	NA	N	N	N	NA	NA	NA	?	NA	?	?	?	?	L
Borba2005§ [26]	BL+CO	Y	N	N	N	?	?	?	?	?	?	?	?	?	?
Brundell2002 [3]	BL	NA	N	N	N	NA	NA	NA	?	NA	?	?	L	?	L
Carmona2008 [4]	BL+CO	Y	N	N	N	?	?	?	?	?	?	?	?	?	?
Chiu1994 [27]	BL	NA	N	N	N	NA	NA	NA	?	NA	?	?	?	?	L
Chiu1995 [28]	BL	NA	N	N	Y	NA	NA	NA	?	NA	?	?	?	?	?
Chiu1996 [29]	BL	NA	N	N	N	NA	NA	NA	?	NA	?	?	?	?	L
deBarros2012† [30]	CO	Y	N	N	N	?	?	?	?	?	?	?	?	?	?
deFreitas2013 [31]	CO	Y	Y	N	N	?	?	?	?	?	?	?	L	?	L
Demyttenaere2006 [32]	BL	NA	Y	N	Y	NA	NA	NA	?	NA	L	?	L	?	L
Demyttenaere2007 [33]	BL	NA	N	Y	N	NA	NA	NA	?	NA	?	?	L	?	L
French1951§ [34]	CO	N	N	N	N	?	?	?	?	?	?	?	?	?	?
Ge2009 [12]	CO	Y	N	N	N	?	L	?	?	?	?	?	L	?	?
Gong2011 [35]	BL+CO	Y	Y	N	Y	L	?	?	?	?	?	?	?	?	?
Guler1998 [5]	BL+CO	N	N	N	N	?	?	?	?	?	?	?	?	?	L
Hashikura1994 [36]	BL	NA	N	N	N	NA	NA	NA	?	NA	?	?	?	?	?
Junghans1997§† [37]	BL	NA	N	N	N	NA	NA	NA	?	NA	?	?	?	?	L
Kacmaz2003 [38]	CO	N	N	N	N	?	?	?	?	?	?	?	?	?	L
Ke2012 [39]	BL+CO	N	Y	N	Y	L	?	?	?	?	?	?	L	?	L
Khoury2008† [40]	CO	Y	Y	N	N	?	?	?	?	?	?	?	L	?	L
Kirsch1994 [41]	BL+CO	N	N	N	N	L	?	?	?	?	?	?	L	?	H
Lee1999† [42]	CO	N	Y	N	N	?	?	?	?	?	?	?	H	?	L
Li2015 [43]§	CO	Y	Y	N	N	L	?	?	?	?	?	?	L	?	L

S1 Table: Individual risk of bias scores

study	control data type	any randomisation	any blinding	sample size calculation	temperature control	groups similar at baseline (selection)	random group allocation (selection)	blinded group allocation (selection)	random housing (performance)	blinded interventions (performance)	blinded outcome assessment (detection)	random outcome assessment (detection)	attrition bias	selective outcome reporting bias	other biases
Lindberg2003 [44]	BL+CO	N	N	N	Y	?	?	?	?	?	?	?	L	?	?
Lindstrom2003a [45]	BL+CO	N	N	N	Y	L	?	?	?	?	?	?	?	?	L
Lindstrom2003b [46]	CO	N	N	N	Y	L	?	?	?	?	?	?	?	?	L
London2000§ [47]	BL	NA	N	N	Y	NA	NA	NA	?	NA	?	?	?	?	?
McDougall1996§ [1]	CO	N	N	N	N	?	?	?	?	?	?	?	L	?	L
Moller2012 [48]	BL+CO	N	N	N	N	?	?	?	?	?	?	?	L	?	L
Naffaa2013 [49]	BL	NA	Y	N	N	NA	NA	NA	?	NA	?	?	?	?	L
Rosin2002 [50]	BL	NA	N	N	N	NA	NA	NA	?	NA	?	?	L	?	L
Saracoglu2013 [51]	CO	Y	N	N	N	L	?	?	?	?	?	?	?	?	?
Schachtrupp2002 [52]	BL+CO	N	Y	N	Y	L	?	?	?	?	?	?	?	?	L
Schachtrupp2005 [53]	BL+CO	N	Y	N	Y	L	?	?	?	?	?	?	?	?	L
Schafer2001 [54]	BL	NA	N	N	Y	NA	NA	NA	?	NA	?	?	H	?	L
Sener2003 [55]	CO	N	N	N	N	?	?	?	?	?	?	?	?	?	L
Shimazutsu2009 [56]	BL+CO	N	N	N	Y	?	?	?	?	?	?	?	H	?	L
Shuto1995 [57]	BL	NA	N	N	N	NA	NA	NA	?	NA	?	?	L	?	L
Tanaka2002 [58]	BL+CO	N	N	N	N	L	?	?	?	?	?	?	L	?	L
Tsugawa1999 [59]	BL	NA	N	N	N	NA	NA	NA	?	NA	?	?	?	?	L
Varshavavskii1967† [60]	BL	NA	N	N	N	NA	NA	NA	?	NA	?	?	?	?	H
Wiesenthal2011§ [61]	BL	NA	N	N	Y	NA	NA	NA	?	NA	?	?	?	?	L
Xu2012§† [13]	BL+CO	Y	N	N	N	?	?	?	?	?	?	?	?	?	L
Yavuz2001[62]	BL+CO	N	N	N	N	?	?	?	?	?	?	?	L	?	L

§not included in meta-analysis due to missing data; †not included in meta-analysis due to post-PnP data only; BL = Δ baseline; CO = separate control group; NA = not applicable; N = no; Y = yes; ? = unclear risk of bias; L = low risk of bias; H = high risk of bias
